# Pole-Like Object Extraction and Pole-Aided GNSS/IMU/LiDAR-SLAM System in Urban Area

**DOI:** 10.3390/s20247145

**Published:** 2020-12-13

**Authors:** Tianyi Liu, Le Chang, Xiaoji Niu, Jingnan Liu

**Affiliations:** 1 GNSS Research Center, Wuhan University, 129 Luoyu Road, Wuhan 430079, China; liutianyi@whu.edu.cn (T.L.); changlesgg@whu.edu.cn (L.C.); xjniu@whu.edu.cn (X.N.); 2 Artificial Intelligence Institute, Wuhan University, 129 Luoyu Road, Wuhan 430079, China; 3 Collaborative Innovation Center of Geospatial Technology, Wuhan University, 129 Luoyu Road, Wuhan 430079, China

**Keywords:** urban environment, pole extraction, neural network, LiDAR, integrated navigation

## Abstract

Vision-based sensors such as LiDAR (Light Detection and Ranging) are adopted in the SLAM (Simultaneous Localization and Mapping) system. In the 16-beam LiDAR aided SLAM system, due to the difficulty of object detection by sparse laser data, neither the grid-based nor feature point-based solution can avoid the interference of moving objects. In an urban environment, the pole-like objects are common, invariant and have distinguishing characteristics. Therefore, it is suitable to bring more robust and reliable positioning results as auxiliary information in the process of vehicle positioning and navigation. In this work, we proposed a scheme of a SLAM system using a GNSS (Global Navigation Satellite System), IMU (Inertial Measurement Unit) and LiDAR sensor using the position of pole-like objects as the features for SLAM. The scheme combines a traditional preprocessing method and a small scale artificial neural network to extract the pole-like objects in environment. Firstly, the threshold-based method is used to extract the pole-like object candidates from the point cloud, and then, the neural network is applied for training and inference to obtain pole-like objects. The result shows that the accuracy and recall rate are sufficient to provide stable observation for the following SLAM process. After extracting the poles from the LiDAR point cloud, their coordinates are added to the feature map, and the nonlinear optimization of the front end is carried out by utilizing the distance constraints corresponding to the pole coordinates; then, the heading angle and horizontal plane translation are estimated. The ground feature points are used to enhance the elevation, pitch and roll angle accuracy. The performance of the proposed navigation system is evaluated through field experiments by checking the position drift and attitude errors during multiple two-min mimic GNSS outages without additional IMU motion constrain such as NHC (Nonholonomic Constrain). The experimental results show that the performance of the proposed scheme is superior to that of the conventional feature point grid-based SLAM with the same back end, especially in congested crossroads where slow-moving vehicles are surrounded and pole-like objects are rich in the environment. The mean plane position error during two-min GNSS outages was reduced by 38.5%, and the root mean square error was reduced by 35.3%. Therefore, the proposed pole-like feature-based GNSS/IMU/LiDAR SLAM system can fuse condensed information from those sensors effectively to mitigate positioning and orientation errors, even in a short-time GNSS denied environment.

## 1. Introduction

An automatic driving system is an unremitting pursuit of intelligent vehicles, and the navigation ability is the essential guarantee for vehicles to successfully complete the automatic driving task safely and accurately. A Global Navigation Satellite System (GNSS), which includes a GPS (Global Positioning System), BDS (BeiDou Navigation Satellite System) [1,2], GLONASS and Galileo, can easily provide the position information of the target for their users. However, in urban environment, due to the existence of buildings and viaducts, low-quality observations, i.e., multi-path signals and NLOS (Non-Line-Of-Sight) signals, are frequently obtained, causing unreliable GNSS positioning results. Although some processes [3] can be absorbed in the navigation solution to improve the positioning, the precision is still insufficient for automatic driving. Therefore, it is difficult to ensure the accuracy of vehicle positioning. In the widely used integrated navigation scheme, data from GNSS and Inertial Measurement Unit (IMU) are fused by a Kalman filter [4] or graph optimization [5,6,7]. However, errors from medium-end and low-end IMU will accumulate rapidly over time [8,9], and the positioning error will also appear after the GNSS measurement quality deteriorates for a period of time. Therefore, the fusion of multisource sensors is the inevitable choice of an automatic driving positioning scheme.

Vision information obtained from a camera is widely used in Simultaneous Localization and Mapping (SLAM) [10,11,12,13,14,15]. Compared to the camera, Light Detection and Ranging (LiDAR) can not only work under different weather and different illumination but, also, get a 3D structure of its surroundings. As an important component of the current automatic driving system or mobile robots, LiDAR can undertake positioning, sensing and mapping tasks, so it has been widely used [16,17,18,19,20,21,22,23,24]. However, researchers always try to use common and concise features to make it. Zhao and Farrell [25] used the laser reflections of 2D LiDAR scanning to detect lines to associate with mapped building planes and then used them as anchors to get the position and orientation of the LiDAR. Im et al. [26] used point cloud data to extract the corner features of buildings to locate vehicles in urban environments. In the process of urban modernization, for the sake of the attractiveness of buildings, many of them in commercial and residential areas use a large amount of glass materials as their walls. A glass material will produce specular reflection and irregular scattering to the electromagnetic wave emitted by LiDAR, which brings remarkable LiDAR measurement errors. In congested areas, moving vehicles such as vans, trucks and buses always provide unreliable surface or edge feature points for LiDAR scan matching. As a result of this, errors of position estimations and orientation determinations are brought about in dynamic environments. However, in urban construction, the infrastructure of traffic poles, streetlight poles and tree planting often comply with certain standards and remain unchanged for a long time. Therefore, in the LiDAR integrated navigation scheme, it is advantageous to carry out short-term unmanned ground vehicle navigation positioning or maintaining a long-term high-definition map by using pole-like objects as a general feature in an urban environment. In Schaefer et al. [27] and Weng et al. [28], the authors use the navigation system with LiDAR to generate the map in advance and then extract the pole-like objects in the map as landmarks. Finally, the effectiveness of global positioning by matching current scanning to extracted landmarks was proven. However, when the vehicle is in an unknown environment or on a rebuilt street, it is impossible to obtain the prebuild map for global localization. In Cabo et al. [29], Rodríguez-Cuenca et al. [30], Yu et al. [31], Zheng et al. [32] and Wu et al. [33], dense and high-ranging precision LiDAR data obtained from a MLS (Mobile Laser Scanner) were used to extract street furniture in a city, and excellent results were shown in these papers. In these survey tasks above, platform vehicles can move slowly, and object extraction can be done in postprocessing. However, in an automatic driving task, only low-cost sensors can be accepted in the system, and the designed algorithms should also work in high-speed driving scenarios in real time. As a consequence, the ability of pole extraction in a real time point and SLAM is necessary for autonomous vehicles. Semantic tasks such as classification, segmentation and the object detection of a point cloud have been researched for years. Qi et al. [34,35] proposed deep-learning methods for point cloud classification. Wu et al. referred to FCN (Full Convolutional Networks) [36]—the segmentation task in the image processing field and the designed Squeezeseg network and its second version [37,38]—to tackle the segmentation problem on a relative dense point cloud. Zhou et al. [39] came up with an idea of using an end-to-end network to detect vehicles, pedestrians and cyclists in a KITTI (Karlsruhe Institute of Technology and Toyota Technological Institute) dataset. Although they have good performance on these tasks, all of them were designed for dense point cloud data, and GPUs are necessary in these algorithms, alongside high-power consumption. To make full use of power in vehicles and obtain navigation results in time, a slim but efficient algorithm should be proposed to extract pole-like objects from the sparse point cloud obtained by less-beam LiDAR like Velodyne VLP-16.

In this paper, we propose and evaluate the navigation and global localization scheme of automatic driving vehicles by using the pole-like objects in an urban environment, such as tree trunks, light poles and traffic poles, as auxiliary information without using a prebuild map. The scheme consists of a pole extraction algorithm by combining a tradition threshold-based method with a slim artificial neural network to consider both the efficiency and accuracy. The sensors of the proposed system include GNSS, IMU and a 16-beam LiDAR.

## 2. Methods

### 2.1. Coordinate Definition

Since three sensors are used in the proposed system, the following several coordinate systems are defined, and all systems follow the right-hand rule:

1. Body coordinate system (b-frame): The coordinate system of the IMU, where the X-axis is pointing right, Y-axis is pointing forward and Z-axis is pointing down.
2. LiDAR coordinate system (l-frame): This coordinate system is defined as “l”, with the X-axis, Y-axis and Z-axis pointing right, forwards and up, respectively.
3. World coordinate frame (w-frame): The coordinate system of the GNSS positioning results, with the initial GNSS position as the origin, the X-axis pointing east, the Y-axis pointing north and the Z-axis pointing up.
4. Map coordinate system (m-frame): Its origin is the position where the SLAM is initialized, and the X-Y plane is the local horizontal plane. The X-axis of the m-frame is parallel to the X-axis of the b-frame at the time when the system is initialized.

### 2.2. Pole Extraction

Pole-like objects are ubiquitous in urban scenarios, and they occur as parts of streetlamps, trees and traffic signs. Due to their invariance under different seasons and weather changes, pole-like objects have become high-quality landmarks for vehicle localization in autonomous driving. To make full use of this reliable and long-lasting feature, pole extraction has aroused great interest in academia, especially in the research field of autonomous vehicle navigation and high-definition map (HD map)-making [40,41,42]. In Song et al. [43], a point cloud is clustered, and then features such as eigen values, principle components of each cluster, are computed. Then, the feature vector is taken as the input of the back-propagation neural network to get the label of the belonging cluster. However, in reality, traffic signs occlude in the crowns of trees, and tree trunks grow in bushes sometimes. In these cases, the point cloud of traffic signs and tree trunks cannot be segmented into an individual cluster precisely, so the cluster might be classified into an incorrect category, resulting in a low recall of the classification task. Taking navigation and localization into account, a novel method of pole extraction is proposed in this paper. To get high recall of the pole extraction, a traditional method is taken to get pole candidates by relaxed thresholds. Then, features of these candidate point clouds are computed and fed into the neural network, to classify the candidate into pole or non-pole objects.

**Figure 1:**
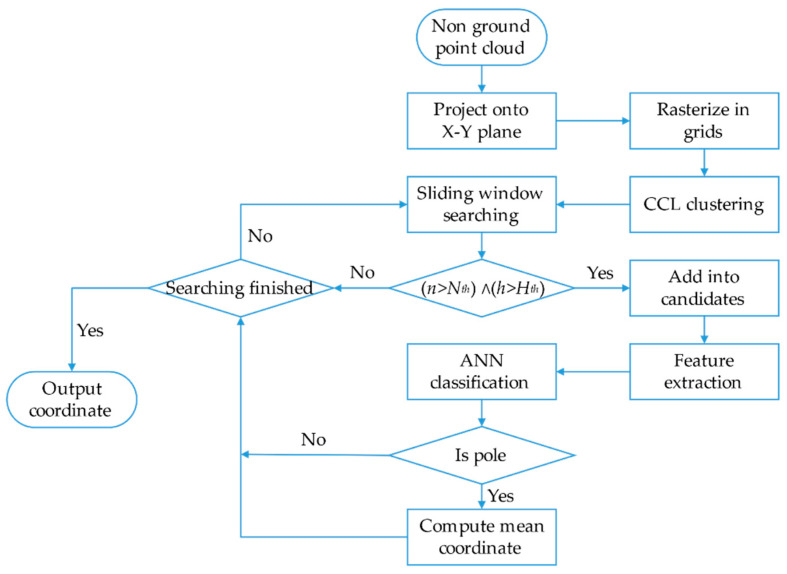
Flow chart of pole candidate extraction from a nonground point cloud. CCL: connected-component-labeling.

Considering the sparsity of the point cloud collected from Velodyne PUCK VLP-16 (called VLP-16 in the following paragraphs) in the vertical direction, point clouds are accumulated over 0.2 s to form one frame or a node. The origin of the frame is the origin of LiDAR at the very beginning of each frame. Ground segmentation is taken as the first step of the pole extraction module, and the method in LeGO-LOAM [44] is adopted in this stage. Then, nonground points are projected onto the X-Y plane and then rasterized into regular 2D grids. A connected-component-labeling (CCL) algorithm is applied to get each individual cluster of projected nonground point clouds. In order to get pole candidates in each cluster, sliding window searching is used; every time, the corner of each gird is set as the searching center; then, with the given radius, a circle cloud is determined by the given radius. If the number of points in the circle exceeds the certain threshold $N_{th}$ and the height is higher than 1.5 m, the set of points will be regarded as a pole candidate. The whole process is shown in Figure 1.

**Figure 2:**
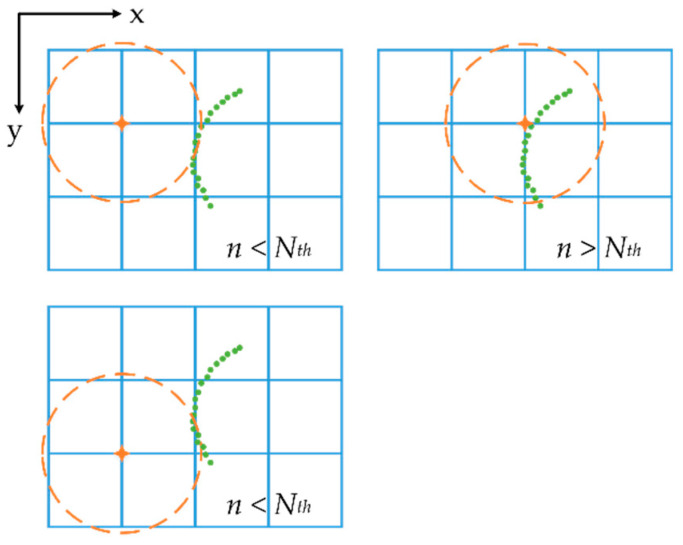
Sliding window searching for pole candidates.

Figure 2 shows the process of pole candidate searching: the blue squares are the rasterized grids, the green points are the local point cloud projected on the X-Y plane and the orange circle is the searching field determined by a given radius whose center is the corner of the grid.

In the previous stages, ground points are removed, and nonground points are clustered into small clusters; therefore, a large number of useless searches are avoided. Multithread techniques can be applied in the sliding window search to ensure the real-time processing of this module.

After pole candidates’ extraction, five features of the candidates are computed: the ratio of the number of pole candidates and the number of its neighborhoods, the minimum value in the z-axis, the maximum of the z-axis, variance of the z-axis and mean intensity. Cross entropy loss is employed as the loss function of the designed neural network in pole and non-pole classification tasks. In Equation (1), N is the amount of samples, $y_{i}$ is the ground truth label of sample i and ${\hat{y}}_{i}$ is the probability predicted by the neural network. In the proposed solution, a shallow artificial neural network with two layers is applied as the classifier. Due to the preprocessing of the threshold-based method, both the performance and generalization are good enough for pole extraction in the navigation task.

(1) $$Loss=-\frac{1}{N}\sum_{i=1}^{N}\left[y_{i}\cdot \log\left({\hat{y}}_{i}\right)+\left(1-y_{i}\right)\cdot \log\left(1-{\hat{y}}_{i}\right)\right],$$

### 2.3. Integrated Navigation Solution

**Figure 3:**
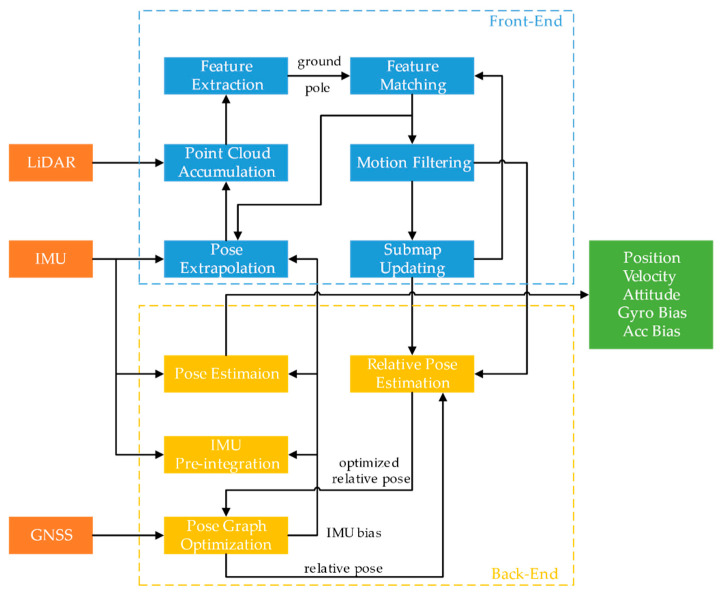
Flow chart of the proposed integrated navigation system. LiDAR: Light Detection and Ranging, IMU: Inertial Measurement Unit and GNSS: Global Navigation Satellite System.

The whole system can be subdivided into the following parts: ground segmentation, pole extraction, pose extrapolation, scan-to-map matching, IMU preintegration and pose graph optimization. The flow chart is shown in Figure 3.

#### 2.3.1. Pose Extrapolation

The IMU here in this system is employed to do pose extrapolation for LiDAR motion compensation and provide an initial guess of the transformation of the sensors. Equations (2)–(5) show the process of pose extrapolation by the output of the IMU.

(2) $$\Delta \tau_{i}=\tau_{i}-\tau_{i-1},$$

(3) $$v_{mb_{i}}^{m}=v_{mb_{i-1}}^{m}+C_{b_{i-1}}^{m}\Delta v_{b_{i-1}b_{i}}^{b_{i-1}}+g^{m}\Delta \tau_{i},$$

(4) $$P_{mb_{i}}^{m}=P_{mb_{i-1}}^{m}+v_{mb_{i-1}}^{m}\Delta \tau_{i}+\frac{1}{2}g^{m}\Delta \tau_{i}{}^{2}+\frac{1}{2}C_{b_{i-1}}^{m}v_{b_{i-1}b_{i}}^{b_{i-1}}\Delta \tau_{i},$$

(5) $$q_{b_{i}}^{m}=q_{b_{i-1}}^{m}⊗q_{b_{i}}^{b_{i-1}}.$$

$\tau_{i}$ in Equation (2) is the ith timestamp of the sensor; $b_{i}$ corresponds to the b-frame at time i; $v_{mb_{i}}^{m}$ is the velocity in the b-frame relative to the m-frame, which projected on the m-frame at time i; $C_{b_{i-1}}^{m}$ is the direction cosine matrix representing the rotation from the b-frame to m-frame; $g^{m}$ is denoted as the gravity in the m-frame; P, v and q are the position, velocity and pose (represented as the quaternion), respectively and the symbol ⊗ is the multiplication of the quaternion.

#### 2.3.2. Feature Matching

In this paper, laser points reflected from ground are assumed to fit well by the same set of plane parameters locally. This supposedly was validated by Shan et al. [44] and Liu et al. [45]. The method of ground segmentation mentioned in Himmelsbach et al. [46] is applied to extract plane feature points on the ground. Next, the voxel filter is applied to reduce the computational cost in the feature-matching process. Note the transformation matrix from the l-frame to the m-frame as $T_{l}^{m}$ composed by the rotation matrix and translation vector and can be written as Equation (6) in a block matrix format:

(6) $$T_{l}^{m}=\left[\begin{array}{cc} C_{l}^{m} & t_{l}^{m}\\ 0_{\left(1\times 3\right)} & 1 \end{array}\right].$$

The inverse matrix of $T_{l}^{m}$ is ${\left(T_{l}^{m}\right)}^{-1}$, which satisfies

(7) $${\left(T_{l}^{m}\right)}^{-1}=T_{m}^{l}=\left[\begin{array}{cc} {\left(C_{l}^{m}\right)}^{T} & -{\left(C_{l}^{m}\right)}^{T}\cdot t_{l}^{m}\\ 0_{\left(1\times 3\right)} & 1 \end{array}\right].$$

The optimized transformation matrix from the l-frame to the m-frame at timestamp i−1 is $T_{l,i-1}^{m}$, and the lever arm and misalign angle between the IMU and LiDAR are denoted as $t_{b}^{l}$ and $C_{b}^{l}$. The transformation $T_{b,i}^{b,i-1}$ from time i to i−1 can be calculated by the output of the IMU. Combined with $t_{b}^{l}$ and $C_{b}^{l}$ mentioned above, the initial guess of the transformation of the l-frame from time i to time i−1, ${\hat{T}}_{l,i}^{l,i-1}$ can be computed as

(8) $${\hat{T}}_{l,i}^{l,i-1}=T_{b}^{l,i}\cdot {\hat{T}}_{b,i}^{b,i-1}\cdot T_{l,i-1}^{b}=\left[\begin{array}{cc} C_{b}^{l} & t_{b}^{l}\\ 0_{\left(1\times 3\right)} & 1 \end{array}\right]\cdot {\hat{T}}_{b,i}^{b,i-1}\cdot \left[\begin{array}{cc} {\left(C_{b}^{l}\right)}^{T} & -{\left(C_{b}^{l}\right)}^{T}\cdot t_{b}^{l}\\ 0_{\left(1\times 3\right)} & 1 \end{array}\right],$$

Then, at time i, the transformation from the l-frame to the m-frame is obtained:

(9) $${\hat{T}}_{l,i}^{m}=T_{l,i-1}^{m}\cdot {\hat{T}}_{l,i}^{l,i-1}.$$

The laser reflection of LiDAR is denoted as

(10) $$p={\left[\begin{array}{ccc} x & y & z \end{array}\right]}^{T},$$

while the homogeneous format of the 3D point is

(11) $${}^{h}p={\left[\begin{array}{cccc} x & y & z & 1 \end{array}\right]}^{T}.$$

To simplify the Equations, the superscript h is omitted, and the nonhomogeneous format of the points is used in the following equations, where the actually homogeneous coordinate is operated with the 4-dimensional transformation matrix. For the purpose of estimating the roll, pitch angle and elevation, feature points in the l-frame are transformed into the m-frame, and then, 5 neighbor feature points $p^{m,j},j\in \left\{1,2,3,4,5\right\}$ are found in the m-frame to fit a plane. The formula of the plane is

(12) Ax+By+Cz+d=0,

where $\sqrt{A^{2}+B^{2}+C^{2}}=1$ and unit normal $n={\left[\begin{array}{ccc} A & B & C \end{array}\right]}^{T}$. Roll, pitch angle and elevation are estimated to minimize the distance between the feature point and fitted plane. However, the feature points too far from the fitted plane would not be absorbed in the cost function. Suppose there are K points that are satisfied with the distance threshold, then the objective function can be written as

(13) $$\underset{\theta_{l,roll}^{m},\theta_{l,pitch}^{m},t_{z}}{\arg\min}\sum_{k=1}^{K}\left|n\cdot \left({\hat{T}}_{l}^{m}\cdot p^{l,k}\right)+d_{k}\right|.$$

In the above Equation (13), $\theta_{l,roll}^{m}$, $\theta_{l,pitch}^{m}$ and $t_{z}$ are the corresponding components in the transformation matrix representing the roll, pitch angle and height translation, and $d_{k}$ is the distance between the kth feature point to its local plane.

On the other hand, point clouds of poles are obtained by the algorithm introduced in Section 2.2. Consequently, the mean of x and y marked as pole feature points can be used to optimize the translation in the x and y directions and yaw angle of the vehicle by minimizing the horizontal distance between correspondences in the m-frame:

(14) $$\underset{\theta_{l,yaw}^{m},t_{l,x}^{m},t_{l,y}^{m}}{\arg\min}\sum_{k=1}^{K}\left\|p^{m,j}-{\hat{T}}_{l}^{m}\cdot p^{l,k}\right\|.$$

#### 2.3.3. Optimization in the Back End

In this paper, the IMU preintegration, GNSS position and relative transformation between the LiDAR nodes and submaps are used as constrains to establish the pose graph for pose optimization in the back end (Chang et al. [47,48]). The variable that is going to be optimized can be written as χ, where $x_{k}$ is composed of the position, velocity, attitude, accelerometer bias and gyroscope bias of the IMU at time $\tau_{k}$. N is the number of nodes, $y_{k}$ is the position and attitude of the submap in the m-frame at $\tau_{k}$ and M is the number of submaps:

(15) $$\begin{array}{l} \chi =\left[x_{1},x_{2},x_{3},\ldots x_{N},y_{1},y_{2},y_{3},\ldots y_{M},P_{wm}^{w},q_{m}^{w}\right]\\ x_{k}=\left[P_{mb_{k}}^{m},v_{mb_{k}}^{m},q_{mb_{k}}^{m},b_{a,k},b_{g,k}\right],k\in \left\{1,2,3,\ldots N\right\}\\ y_{k}=\left[P_{ms_{k}}^{m},q_{ms_{k}}^{m}\right],k\in \left\{1,2,3,\ldots M\right\} \end{array}$$

The cost functions of pose graph optimization are Equation (16)

(16) $$\underset{\chi }{\arg\min}\left\{\begin{array}{l} \sum_{i\in \alpha }E_{GNSS}^{2}\left(x_{i},P_{wm}^{w},q_{m}^{w},p_{g_{i}}^{w},l_{g}^{b},\sigma_{p}\right)+\\ \sum_{\begin{array}{l} i\in \beta ,\\ j\in \kappa \end{array}}E_{LiDAR}^{2}\left(x_{i},y_{j},P_{bl}^{b},q_{b}^{l},P_{s_{j}l_{i}}^{s_{j}},q_{l_{i}}^{s_{j}},\sigma_{ij}\right)+\\ \sum_{i=1}^{N-1}E_{IMU}^{2}\left(x_{i},x_{i+1},z_{i+1}^{i},\sigma_{z}\right) \end{array}\right\}.$$

$E_{GNSS}^{2}\left(\cdot \right)$, $E_{LiDAR}^{2}\left(\cdot \right)$ and $E_{IMU}^{2}\left(\cdot \right)$ are the cost functions of the GNSS, LiDAR and IMU, respectively. $p_{g_{i}}^{w}$ is the positioning result in the w-frame, $l_{g}^{b}$ is the lever arm between the GNSS antenna and IMU (pointing to the phase center of the antenna from the centroid of the IMU), $\sigma_{p}$ is the standard deviation of $p_{g}^{w}$, α is the nodes set with the GNSS positioning result, $P_{s_{j}l_{i}}^{s_{j}}$ and $q_{l_{i}}^{s_{j}}$ are the relative position and attitude of the node $l_{i}$ and the submap $s_{j}$, $\sigma_{ij}$ is the standard deviation of $P_{s_{j}l_{i}}^{s_{j}}$ and $q_{l_{i}}^{s_{j}}$, β is the nodes set, κ is the submaps set, $z_{i+1}^{i}$ is the preintegration result between the $t_{i}$ node and the $t_{i+1}$ node and $\sigma_{z}$ is the standard deviation of z. $P_{bl}^{b}$ and $q_{b}^{l}$ are the extrinsic parameters of LiDAR and IMU, including the lever arm (pointing to the center of the laser transmitter from the centroid of the IMU) and misaligned angle (projected into the b-frame). According to Chang et al. [47], the cost functions of GNSS and LiDAR can be obtained:

(17) $$\begin{array}{ccc} E_{GNSS}^{2}\left(x_{i},P_{wm}^{w},q_{m}^{w},p_{g_{i}}^{w},l_{g}^{b},\sigma_{p}\right) & = & e{\left(x_{i},P_{wm}^{w},q_{m}^{w},p_{g_{i}}^{w},l_{g}^{b}\right)}^{T}{\left(\sigma_{p}^{2}\right)}^{-1}e\left(x_{i},P_{wm}^{w},q_{m}^{w},p_{g_{i}}^{w},l_{g}^{b}\right)\\ e\left(x_{i},P_{wm}^{w},q_{m}^{w},p_{g_{i}}^{w},l_{g}^{b}\right) & = & C_{m}^{w}\left(C_{b_{i}}^{m}l_{g}^{b}+p_{mb_{i}}^{m}\right)+P_{wm}^{w}-p_{g_{i}}^{w}\\ E_{LiDAR}^{2}\left(x_{i},y_{j},P_{bl}^{b},q_{l}^{b},P_{s_{j}l_{i}}^{s_{j}},q_{l_{i}}^{s_{j}},\sigma_{ij}\right) & = & e{\left(x_{i},y_{j},P_{bl}^{b},q_{l}^{b},P_{s_{j}l_{i}}^{s_{j}},q_{l_{i}}^{s_{j}}\right)}^{T}{\left(\sigma_{ij}^{2}\right)}^{-1}e(x_{i},y_{j},P_{bl}^{b},q_{l}^{b},P_{s_{j}l_{i}}^{s_{j}},q_{l_{i}}^{s_{j}}),\\ e\left(x_{i},y_{j},P_{bl}^{b},q_{l}^{b},P_{s_{j}l_{i}}^{s_{j}},q_{l_{i}}^{s_{j}}\right) & = & \left[\begin{array}{c} {\left(C_{s_{j}}^{w}\right)}^{-1}\left[p_{mb_{i}}^{m}+C_{b_{j}}^{w}p_{bl}^{b}-p_{ms_{j}}^{m}\right]-p_{s_{j}l_{i}}^{s_{j}}\\ {\left[{\left(q_{b_{i}}^{m}⊗q_{l}^{b}\right)}^{-1}⊗q_{s_{j}}^{m}⊗q_{l_{i}}^{s_{j}}\right]}_{xyz} \end{array}\right]\\ E_{IMU}^{2}\left(x_{k-1},x_{k},z_{t_{k}}^{t_{k-1}},\sigma_{z}\right) & = & e{\left(x_{k-1},x_{k},z_{t_{k}}^{t_{k-1}}\right)}^{T}{\left(\sigma_{z}^{2}\right)}^{-1}e\left(x_{k-1},x_{k},z_{t_{k}}^{t_{k-1}}\right) \end{array}$$

where e(⋅) is the residual function, and ${\left[q\right]}_{xyz}$ represents the rotation vector equivalent to q.

(18) $$\begin{array}{l} \alpha_{b_{k-1}}^{b_{k-1}}=0,\beta_{b_{k-1}}^{b_{k-1}}=0,q_{b_{k-1}}^{b_{k-1}}=I\\ \beta_{b_{i}}^{b_{k-1}}=\beta_{b_{i-1}}^{b_{k-1}}+C_{b_{i-1}}^{b_{k-1}}v_{b_{i-1}b_{i}}^{b_{i-1}}\\ \alpha_{b_{i}}^{b_{k-1}}=\alpha_{b_{i-1}}^{b_{k-1}}+\frac{1}{2}C_{b_{i-1}}^{b_{k-1}}v_{b_{i-1}b_{i}}^{b_{i-1}}\Delta \tau_{i}\\ q_{b_{i}}^{b_{k-1}}=q_{b_{i-1}}^{b_{k-1}}⊗q_{b_{i}}^{b_{i-1}} \end{array}$$

$\alpha_{b_{i}}^{b_{k-1}}$, $\beta_{b_{i}}^{b_{k-1}}$ and $q_{b_{i}}^{b_{k-1}}$ are the increments of position, velocity and attitude calculated from the IMU preintegration, only related to the output of the IMU, being independent to the initial state. $\tau_{i}$ is some timestamp between $\left[\tau_{k-1},\tau_{k}\right]$.

## 3. Experiments

**Figure 4:**
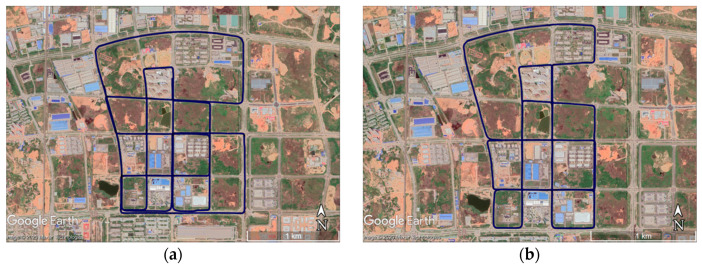
Trajectories of the tests projected on Google Earth: (**a**) trajectory of test #1 and (**b**) trajectory of test #2.

The experimental data was collected in a suburb area in Wuhan City in an open-sky environment to ensure a high-quality GNSS RTK (Real Time Kinematic) signal and the precision of the reference system. The RTK base station was set several kilometers away from the experimental field. The trajectories of the data collection projected on Google Earth are shown in Figure 4.

In order to mitigate the correlation between two neighbor outages, the interval between them is set as 180 s, and each outage lasts for 120 s.

**Figure 5:**
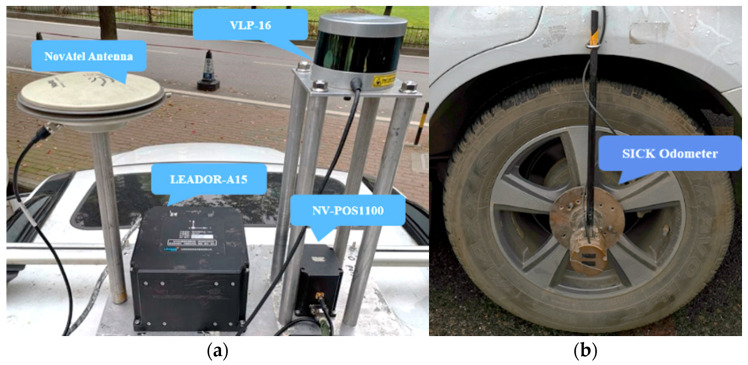
Experimental equipment: (**a**) equipment installed on the roof of the testing vehicle and (**b**) odometer.

The reference system of this experiment consisted of IMU-A15 IMU—a high-level tactical-grade IMU with a laser gyroscope produced by the Leador Spatial Information Technology Corporation (Wuhan, China), a SICK-DFS60E-BECM02048 odometer (SICK, Waldkirch, Germany) and GNSS antenna from NovAtel (Calgary, AB, Canada). The experimental system was equipped with NV-POS1100—a quasi-tactical grade MEMS IMU (Sai MicroElectronics Inc., Beijing, China), Velodyne PUCK VLP-16 (Velodyne Lidar Inc., San Jose, CA, USA) and a common GNSS antenna with a reference system. The IMU-A15 IMU or SICK odometer were not used in the testing system. Figure 5 shows the equipments of the field test.

The specified parameters of the IMUs are in Table 1.

The sampling rate of each sensor is shown in Table 2.

## 4. Results and Discussion

The performance pole classifications and integrated navigation algorithms will be discussed separately.

In the training stage of the pole candidate classifications, each frame of the point cloud was accumulated by the pose obtained from the front end of Chang et al. [47]; then, the ground was removed, and points were clustered by the method in Section 2.2. Finally, the samples were generated by manual labeling. There were 813 samples in total, with 394 poles and 419 nonpoles. Eighty percent of the samples were utilized as a training set, while the rest of samples were used for testing. To be general, the frames where pole candidates were extracted were expected to cover a diversity of scenarios, including crossroads and straight roads and contain less layout with each other. As the velocity of the vehicle was about 10 m/s and the maximum range of LiDAR detection was about 100 m, a 10-s interval was set to get a sequence of LiDAR frames. Accuracy, precision, recall and false positive rate (FPR) were applied to evaluate the performance of the training neural network. The performance of the pole classification neural network is shown in Table 3. The average time consumption of this module is about 0.03 s—much less than the time interval of LiDAR data accumulation as mentioned in Section 2.2. It is also worth mentioning that the training data and test data were collected in July 2019, while the data tested for the performance of the navigation module was collected in April 2019 in the next section. Therefore, the generalization and effectiveness of the pole extraction module will be validated.

**Figure 6:**
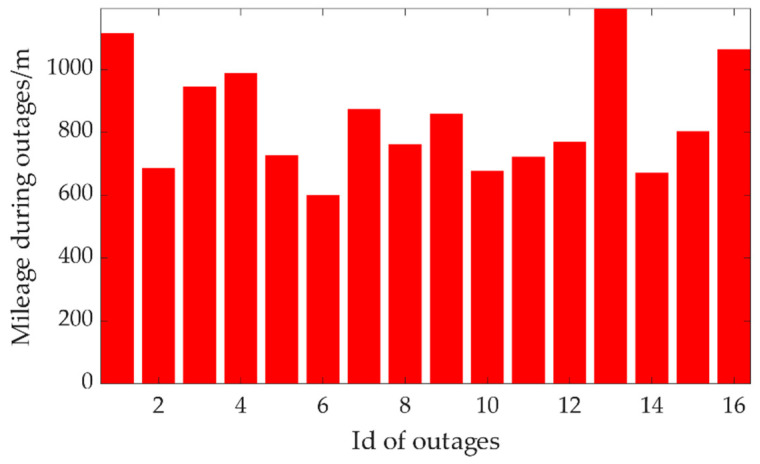
Mileage of the vehicle during mimic GNSS outages.

In the integrated navigation module, 16 mimic GNSS outages in two series of data were set to assess the performance. The shortest mileage of the testing platform during these outages was about 600 m, and the longest was about 1193 m in the plane, as shown in Figure 6.

**Figure 7:**
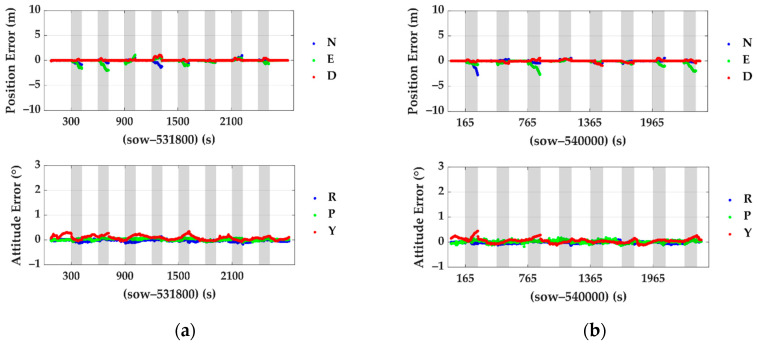
Position and attitude errors of the proposed integrated navigation system during GNSS outages: (**a**) errors of test #1 and (**b**) errors of test #2. The titles N, E and D represent the north, east and down directions, respectively, and R, P and Y mean roll, pitch and yaw.

**Figure 8:**
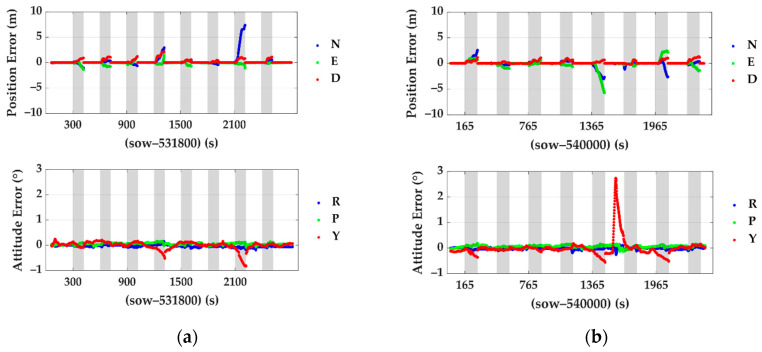
Position and attitude errors of the Feature Point Grid-Based Simultaneous Localization and Mapping (FPG SLAM) during the GNSS outages: (**a**) errors of test #1 and (**b**) errors of test #2.

The benchmark method in Chang et al. [47] (Feature Point Grid-Based SLAM, called FPG SLAM in the rest of this paper) are analyzed here to make a comparison with the proposed method. Position errors and attitude errors during outages of the proposed method and benchmark are statistically analyzed. The details of the pose estimation errors and localization errors in every outage period are plotted in Figure 7 and Figure 8. The grey spans in those figures mark the mimic GNSS outage periods.

The statistical results are also demonstrated in Table 4. The top rows are the statistic results of the benchmarked system. The bottom two rows are the results of the proposed method. The titles N, E and D represent the north, east and down directions, respectively, and R, P and Y mean roll, pitch and yaw. To provide an intuitive impression, the position errors of the traditional integrated navigation system composed of GNSS/IMU during the same outages are also calculated. The RMS (root mean square) errors in the north, east and down directions are 33.22 m, 23.47 m and 3.65 m, respectively, which are several times larger than the drift of the proposed method and FPG SLAM solution.

**Figure 9:**
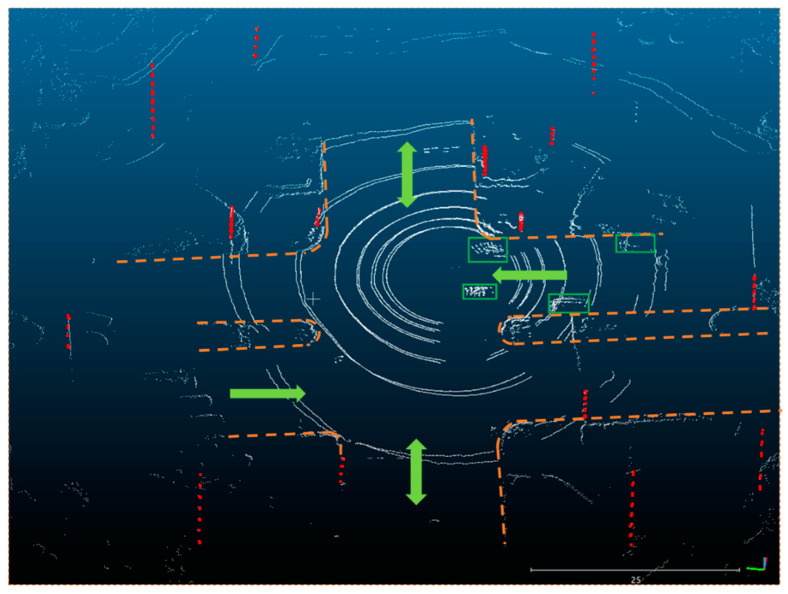
Point cloud at the 533,020th second of the week (in the 4th outage in Figure 8a). The red points are poles extracted by the algorithm mentioned above in Section 2.2; points in green boxes are laser reflections of the surrounding cars, and the point clouds are shown in white. The light green arrows are the directions of roads, and the orange dotted lines are the borders of accessible area.

Compared to FPG SLAM, both the maximum error and root mean square error of the position and attitude of the proposed method are smaller. In detail, the position error RMS was reduced by 61.6% and 31.8% in the north and east. The yaw angle error RMS was reduced by 35.3%. In general, it is more robust in some cases than the FPG SLAM, especially in congested environments where the target vehicle (or the LiDAR) is surrounded by other cars and pole-like objects are rich in the environment. Take the fourth and seventh outages in test #1, as shown in Figure 8a. The following Figure 9 shows the point cloud accumulated at around the 533,020th second of the week (contained in outage 4 in test #1). The white points are the point cloud, and the poles extracted by the pole extraction module are the red ones. The green boxes show the positions of the surrounding vehicles. The light green arrows show the directions of the roads. The orange dotted lines show the borders of the accessible areas of vehicles. In FPG SLAM, the feature points are extracted from the surrounding cars’ reflections, while they deteriorate the estimation of the heading angle and position since the relative motion.

**Figure 10:**
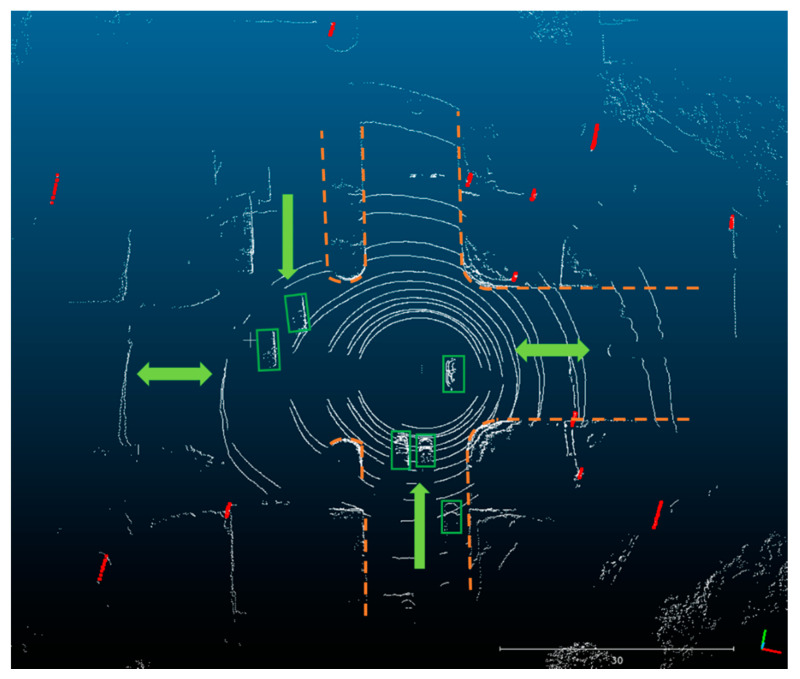
Point cloud at the 533,920th second of the week (in the 7th outage in Figure 8a). Cars in this environment were distributed in different distances and directions, and there existed relative motions between the neighbor cars and the testing vehicle. The color scheme is the same as in Figure 9.

At about the 533,920th second of the week (which was contained in outage 7 in test #1), the testing vehicle was surrounded by several cars at a crossroad, as shown in Figure 10. The feature points obtained from the laser reflections of the cars damaged the solutions of the position and attitude optimizations.

**Figure 11:**
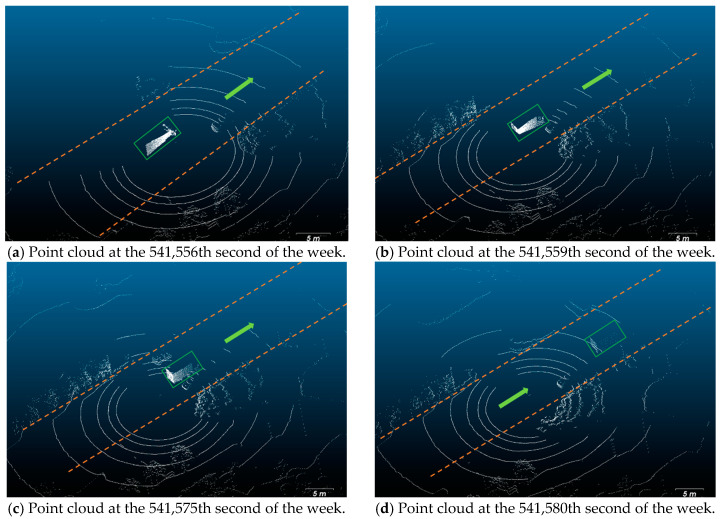
Point cloud collected when an abnormal heading angle error occurred. The laser reflections from the carriage of a van are in the green box. The light green arrow in each figure shows the driving direction of the vehicles, and the orange dotted lines show the borders of the road. The van was at the front-left of the experimental vehicle. (**a**–**d**) show the point cloud at different times, and they disappeared in sequence.

In the FPG SLAM solution, there are a series of salient yaw errors in test #2, as shown in Figure 8b. Tracing back to the real case, the relative position of the testing vehicle and the van changed over time in four stages, and four figures are taken as examples here in Figure 11:

1. The testing vehicle stopped behind a car, and a van got closer and closer to it from the left-behind (shown in Figure 11a).
2. The van passed the testing vehicle, and the tail of the carriage appeared in sight (shown in Figure 11b).
3. The van slowed down and then stopped at the left-front of the testing vehicle for a while (shown in Figure 11c).
4. Both of them restarted moving, and the van disappeared gradually (shown in Figure 11d).

**Figure 12:**
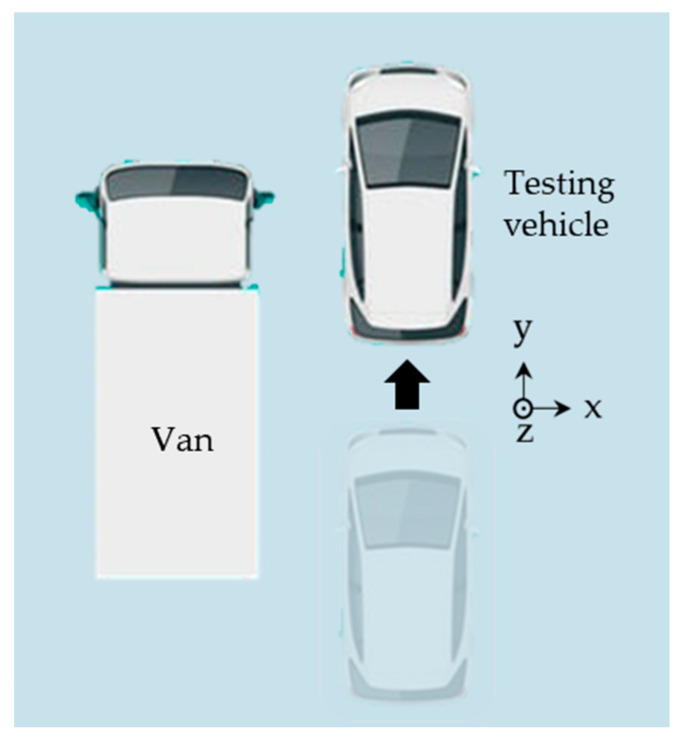
Relative position and motion of the testing vehicle and the neighbor van in a bird eye’s view. The coordinates in the figure show the direction of each axis of the LiDAR. The testing vehicle moved nearly along the y-axis, as the misaligned angle between the vehicle and LiDAR was very small.

The relative position of the testing vehicle and the van is shown in Figure 12.

**Figure 13:**
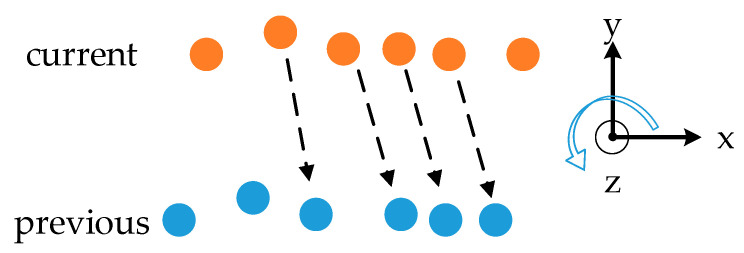
Plane feature points matched to their corresponding ones extracted from the previous LiDAR frame. The orange points are the plane feature points in the current frame, and the blue dots are the plane feature points in the previous frame or feature map. The black dotted line shows some of the correspondence of the feature points. The coordinates on the right show the direction of each axis of the l-frame.

In this paper, we defined the angle error as

(19) $$\Delta \theta_{yaw}=\theta_{yaw}-{\hat{\theta }}_{yaw},$$

where $\Delta \theta_{yaw}$ is the error of the yaw angle, $\theta_{yaw}$ is the ground truth, which increases clockwise and ${\hat{\theta }}_{yaw}$ is the estimation of the yaw angle. A large amount of plane feature points were extracted from the laser reflections of the carriage tail of the van. In stage 2, the distance between the van and the vehicle was too close to make a drifted plane position estimation, while, to match the surface points to their corresponding previous LiDAR frames, the testing vehicle was estimated to turn left, resulting in a smaller yaw angle, as shown in Figure 13.

Thus, positive yaw angle errors appeared in this period of time. In these cases, the LiDAR was surrounded by vehicles with smooth surfaces, such as vans, buses and trucks, along with relative motions among them so that plane feature points were extracted from those moving objects, resulting in bad constraints being added to the optimization problem. In a congested urban area, it is more likely that vans and trucks appear within sight of the LiDAR, causing a wrong estimation of orientation or position of the vehicles.

## 5. Conclusions

To handle the navigation problem in a GNSS-denied environment and take advantage of an urban infrastructure, this paper explored a pole extraction method using sparse LiDAR data and proposed a corresponding robust integrated navigation system composed of GNSS/IMU/LiDAR in an urban area. Experiments were carried out to evaluate the effectiveness of each module in the proposed solution, including the pole extraction module and integrated navigation module. In the validation of the pole extraction module, more than 800 samples were labeled manually. There were 80% of them were used for training a neural network, and 20% of them were used as the test set. The accuracy, precision, recall and FPR of the test set were 91.3%, 88.7%, 93.2% and 10.3%, respectively. The validity of the pole extraction module was verified in the navigation module, which took the coordinates of the poles as the feature points to do scan matching and then posed an optimization. In the integrated navigation module, a benchmark solution—FPG SLAM, as mentioned above, was demonstrated to make a comparison with the proposed method. The pole-based GNSS/IMU/LiDAR method outperformed the FPG SLAM integrated navigation system in terms of the position estimation and attitude estimation during the mimic GNSS outages. Compared with FPG SLAM, the position error of the proposed method was reduced by 61.6% and 31.8% in the north and east, and the yaw angle error RMS was reduced by 35.3%, especially in congested areas such as the crossroad, and the pole-based LiDAR SLAM showed its robustness and reliability in these cases. For future works, more features extracted from an urban infrastructure will be used to complement the insufficiency of the pole-like objects in some areas and enhance the performance of LiDAR-based multi-sensor SLAM.

## Figures and Tables

**Table 1:** Basic parameters of the Inertial Measurement Unit (IMU)-A15 (reference truth) and NV-POS1100.

IMU	Gyroscope		Accelerometer	
	Bias Instability (°/h)	Random Walk Noise (°/√h)	Bias Instability (mGal)	Random Walk Noise (m/s/√h)
IMU-A15	0.027	0.003	15	0.03
NV-POS1100	10	0.20	1000	0.18

**Table 2:** Sampling rate of each sensor in the data collection. GNSS: Global Navigation Satellite System.

Sensor	IMU-A15	NV-POS1100	SICK	GNSS Receiver	VLP-16
Sampling Rate	200 Hz	200 Hz	200 Hz	1 Hz	10 Hz

**Table 3:** Performance of the artificial neural network (ANN) in the pole/nonpole classification task. FPR: false positive rate.

Accuracy	91.3%
Precision	88.7%
Recall	93.2%
FPR	10.3%

**Table 4:** Statistical results of the navigation errors obtained from different solutions during mimic GNSS outages. N, E and D indicate the position errors in the north, east and down, while R, P and Y represent the roll, pitch and yaw errors. FPG SLAM: Feature Point Grid-Based Simultaneous Localization and Mapping.

Solution		Position Error (m)			Relative Plane Error	Attitude Error (°)		
		N	E	D		R	P	Y
FPG SLAM	RMS	2.37	1.79	1.08	0.26%	0.12	0.14	0.34
	MAX	7.37	5.69	2.14		0.18	0.19	0.82
Proposed Method	RMS	0.91	1.22	0.53	0.16%	0.10	0.09	0.22
	MAX	2.69	2.64	1.10		0.14	0.12	0.45
